# Assessment of the Physicochemical and Heavy Metal Qualities of Rooftop Harvested Rainwater in a Rural Community

**DOI:** 10.1002/gch2.201700011

**Published:** 2017-07-17

**Authors:** Isoken Henrietta Igbinosa, Isoken Tito Aighewi

**Affiliations:** ^1^ Department of Environmental Management and Toxicology Faculty of Life Sciences University of Benin Benin City 300283 Nigeria

**Keywords:** chemical quality, harvested rainwater, health risk, heavy metals, rainwater quality, water standards

## Abstract

This study is conducted to assess the quality of harvested rainwater. Rooftop rainwater samples are collected between April and September 2015 from Ugbihioko village near Benin City, Nigeria. Heavy metal concentration and physicochemical quality are determined with the use of standard analytical techniques for water quality, and the results are compared to the World Health Organization (WHO) acceptable limits for drinking water. Of the different water quality parameters, the results show that temperature is within WHO drinking water standards for all locations, but pH, turbidity, sulfate, chloride, and nitrate concentrations vary considerably and do not meet the standards for all locations. Regarding the maximum acceptable concentration (MAC), electrical conductivity is well below the MAC for all cases; the heavy metals copper and iron are above the MAC for all cases; the light metals sodium and potassium are below the MAC for all cases. Lead is above the MAC for all locations, except for in one location; and selenium varied, with some sites having selenium concentrations above the MAC. The results from this study show that public health education or advising is vital for mitigating the possible risks that can be linked to the use of harvested rainwater without treatment.

## 1 Introduction

Rainwater harvesting is the process of collecting and storing rainwater to be utilized for domestic and nondomestic water use by households.^1^ At present, the majority of urban and rural people use harvested rainwater for diverse purposes, including drinking.^2^ Universally, rainwater harvesting is recognized as an alternative water source by several organizations.^3^, ^4^, ^5^ Rainwater harvesting provides water for local settlements in semi‐arid regions of nations around the globe. Evidence shows that in Tanzania, ≈50% of the population depends on rainwater harvesting as their main source of water.^6^ However, no national or international guidelines exist regarding routine analysis and monitoring of rainwater, although potential users should understand the possible threats and hazards associated with harvested rainwater. In a few cases, local authorities have formulated guidelines for the use of harvested rainwater. In Austin, Texas, for example, the Texas Water Development Board collaborated with other investors in 2005 to produce a Rainwater Harvesting Manual that has been recently published as the third edition. This manual aims to instruct the populace on the potential use and application of harvested rainwater.^7^

Poor sanitation and inadequate water supplies impact the socioeconomic status of individuals and communities.^8^, ^9^, ^10^ Several countries around the world rely on harvested rainwater as a vital means of potable water, particularly in rural areas.^11^ The specific health hazards linked to the use of untreated rainwater have yet to be identified. Regulations for the use of rainwater in developing countries, such as in Africa, have yet to be specified.^12^ To be used as an alternative source of water to help mitigate water crises, harvested rainwater must be quantitatively and qualitatively screened.^13^ The quality of harvested rainwater, however, is dependent on several variables or factors, such as atmospheric conditions, the proximity of pollution sources, the maintenance quality of water reservoirs, the type of catchment area and the structure of the storage system.^12^ In addition, increased warnings about the composition of chemical constituents in potable water, especially metals and metalloids, are needed.^14^ In developed countries, there is evidence of harmful concentrations of certain chemical constituents in water,^15^, ^16^ giving rise to controversial definitions of maximum acceptable concentration (MAC) figures for some elements of ground water, surface water, and spring water, resulting in biological and chemical research and governmental debates in many countries^17^ due to their health implications.

The availability of potable water has been a major challenge in most rural and urban areas of Nigeria, a phenomenon related to poverty. Similar scenarios are evident in many other developing areas around the globe.^18^ This has resulted in an overreliance on untreated supplemental sources for domestic and drinking water, such as manmade wells, rivers, and harvested rainwater, which have associated health risks. The associated health risks with the use of harvested rainwater remain a vital concern in rainwater harvesting systems, as chemical pollutants have been reported in rainwater tanks.^19^, ^20^, ^21^, ^22^ Water from rainwater tanks can pose potential health risks, particularly when used as drinking water, due to possible contamination from particulate matter, heavy metals or pathogens from atmospheric deposition, roofing materials, or collection systems. Previous reports on quality assessments of rainwater have been documented from Palestine,^23^ Greece,^24^, ^25^ the Netherlands,^26^ Canada,^27^ the Mediterranean,^28^ Spain,^29^ Australia,^30^, ^31^, ^32^, ^33^ the USA,^34^ France,^35^ South Africa,^36^ Jordan,^37^ South Korea,^38^ and Tanzania,^39^ where sampling location, weather conditions, and agricultural, industrial and urban activities have been reported to significantly impact rooftop harvested rainwater. This risk is particularly accentuated in Nigeria where no water quality regulation or advisory exists for guiding rooftop harvested rainwater. Consequently, our study was initiated to assess the quality of harvested rainwater used for potable and nonpotable practices in a rural community. In our study, we assess the physicochemical quality and heavy metal concentration of rooftop harvested rainwater in a rural community in Benin City, Edo State, Nigeria.

## 2 Results and Discussion

The results of this study provide baseline data on the physicochemical quality and indicators of heavy and light metals in harvested rainwater from a rural community, with reference to the World Health Organization (WHO) standard for water quality.^40^

### 2.1 Physicochemical Indicators

Physicochemical results from the collected rainwater samples are presented in **Table** **1**. The pH of water governs the solubility and biological accessibility of elemental constituents in the water, such as nutrients and heavy metals.^41^ The pH levels of the collected rainwater across the studied household sites were between 5.29 ± 2.01 and 9.79 ± 0.29, indicating that rain is not strongly acidic in the study area. There was a significant difference between the pH values across the sampled household sites (*p* < 0.01). There was no significant difference between the pH levels and temperature (*p* > 0.01) of the collected rainwater samples. The WHO^40^ recommends that the pH of potable water range between 6.5 and 8.5, and the majority of households did not meet this recommendation (Table 1). Drinking water with a pH <4 and >11 can have harmful effects on consumer health. The pH levels of 6.5 or lower can have a corrosive effect, such as in rainwater samples.^42^ Previous studies have documented a pH range of 6–8.5 in rainwater.^43^, ^44^, ^45^

**Table 1:** Physicochemical properties of the collected rainwater samples

Sample location	Physicochemical variables (mean ± SD)a)											
	pH	Temp [°C]	Turbidity [NTU]	TDS [mg L^−1^]	EC [μS cm^−1^]	DO [mg L^−1^]	BOD_5_ [mg L^−1^]	COD [mg L^−1^]	PO_4_ [mg L^−1^]	SO_4_ [mg L^−1^]	Cl^−^ [mg L^−1^]	NO_3_ [mg L^−1^]
Site 1	7.89 ± 1.90	29.50 ± 1.10	3.91 ± 2.10	63.53 ± 5.01	5.32 ± 1.01	4.28 ± 0.25	2.82 ± 0.05	6.93 ± 0.02	0.46 ± 0.01	6.30 ± 0.44	nd	0.42 ± 0.01
Site 2	7.55 ± 0.15	30.25 ± 0.91	3.55 ± 0.12	74.75 ± 3.82	8.75 ± 1.58	3.95 ± 0.52	1.59 ± 0.15	16.54 ± 1.05	0.58 ± 0.04	3.86 ± 0.81	14.50 ± 0.15	0.56 ± 0.08
Site 3	5.46 ± 1.21	29.51 ± 0.20	6.95 ± 2.01	36.80 ± 0.91	3.36 ± 0.25	5.21 ± 0.49	1.12 ± 0.01	16.22 ± 1.25	0.92 ± 0.01	4.73 ± 0.12	15.30 ± 0.12	0.78 ± 0.20
Site 4	5.55 ± 1.01	29.85 ± 0.79	6. 69 ± 1.58	53.56 ± 2.58	6.73 ± 0.32	3.78 ± 0.37	1.87 ± 0.20	30.26 ± 4.25	0.74 ± 0.12	2.30 ± 0.14	nd	0.78 ± 0.05
Site 5	5.50 ± 0.25	30.22 ± 0.12	4.86 ± 1.42	25.70 ± 2.71	16.25 ± 5.01	4.13 ± 0.25	2.13 ± 0.01	26.51 ± 3.52	0.84 ± 0.10	9.58 ± 0.10	nd	0.61 ± 0.03
Site 6	7.85 ± 0.31	29.52 ± 0.09	7.24 ± 2.15	33.50 ± 4.01	4.58 ± 0.50	5.06 ± 0.21	2.06 ± 0.01	24.52 ± 2.51	1.84 ± 0.02	4.89 ± 0.65	nd	0.38 ± 0.01
Site 7	8.95 ± 0.12	28.45 ± 0.23	2.81 ± 0.17	19.45 ± 5.21	3.82 ± 0.28	4.24 ± 0.15	1.46 ± 0.01	8.50 ± 2.45	0.68 ± 0.07	3.16 ± 0.55	15.60 ± 0.25	0.29 ± 0.02
Site 8	5.65 ± 0.06	27.95 ± 0.20	2.50 ± 0.48	16.15 ± 0.57	3.37 ± 0.21	3.43 ± 0.10	2.35 ± 0.00	10.25 ± 1.21	0.62 ± 0.01	8.92 ± 0.16	nd	0.48 ± 0.01
Site 9	5.70 ± 0.92	30.35 ± 0.18	1.81 ± 1.08	24.85 ± 0.05	2.91 ± 0.05	4.09 ± 0.15	1.94 ± 0.15	4.32 ± 1.52	0.58 ± 0.01	2.67 ± 0.47	18.10 ± 1.25	0.87 ± 0.01
Site 10	8.75 ± 0.18	27.45 ± 0.25	1.70 ± 0.05	6.95 ± 1.15	2.89 ± 0.04	3.93 ± 0.19	1.51 ± 0.31	4.42 ± 2.10	0.42 ± 0.05	7.09 ± 0.85	nd	0.57 ± 0.02
Site 11	6.95 ± 2.01	28.22 ± 1.25	1.18 ± 0.14	55.60 ± 3.48	7.15 ± 0.18	3.60 ± 0.10	2.25 ± 0.52	4.55 ± 1.92	0.85 ± 0.01	7.98 ± 0.27	14.80 ± 0.11	0.96 ± 0.04
Site 12	7.81 ± 0.31	27.94 ± 0.15	1.42 ± 0.25	76.15 ± 4.52	33.69 ± 6.42	3.15 ± 0.25	2.24 ± 0.82	5.96 ± 2.01	0.23 ± 0.01	8.64 ± 0.68	27.50 ± 0.21	0.34 ± 0.01
Site 13	9.56 ± 0.19	27.10 ± 0.98	1.15 ± 0.05	45.80 ± 2.46	6.52 ± 0.18	3.26 ± 0.31	2.15 ± 0.00	4.37 ± 1.40	0.75 ± 0.05	5.52 ± 0.25	13.87 ± 1.25	0.91 ± 0.05
Site 14	8.95 ± 0.75	28.90 ± 1.41	1.10 ± 1.21	40.25 ± 1.52	5.28 ± 0.95	3.75 ± 0.35	1.65 ± 0.10	4.10 ± 1.21	0.69 ± 0.02	4.35 ± 0.23	11.95 ± 1.01	0.89 ± 0.02
Site 15	5.85 ± 1.05	30.21 ± 0.91	1.05 ± 0.01	31.50 ± 2.43	2.91 ± 0.21	4.23 ± 0.10	1.42 ± 0.20	3.87 ± 1.02	0.58 ± 0.05	6.82 ± 0.21	11.25 ± 1.20	0.74 ± 0.02
Site 16	8.89 ± 2.05	28.65 ± 0.35	1.30 ± 1.62	62.49 ± 2.59	9.46 ± 0.53	3.43 ± 0.21	2.90 ± 0.01	4.94 ± 1.08	0.14 ± 0.01	9.21 ± 0.43	17.65 ± 0.92	0.25 ± 0.01
Site 17	5.29 ± 2.01	28.72 ± 2.02	1.25 ± 0.01	58.25 ± 2.71	8.32 ± 1.05	3.52 ± 0.02	2.42 ± 0.00	4.81 ± 1.09	0.95 ± 0.05	8.31 ± 0.33	15.45 ± 0.58	0.83 ± 0.02
Site 18	5.68 ± 1.21	29.62 ± 1.89	1.35 ± 0.05	67.03 ± 3.82	11.91 ± 3.45	3.23 ± 0.05	2.65 ± 0.02	5.31 ± 0.18	0.16 ± 0.01	5.52 ± 0.55	17.61 ± 0.24	0.95 ± 0.05
Site 19	9.79 ± 0.29	27.21 ± 0.61	1.28 ± 0.04	59.95 ± 3.46	9.52 ± 1.57	3.51 ± 0.90	2.44 ± 0.05	4.72 ± 0.95	0.13 ± 0.01	7.40 ± 0.41	15.95 ± 1.25	0.41 ± 0.01
Site 20	5.90 ± 1.25	29.55 ± 1.05	1.20 ± 0.20	57.80 ± 4.52	7.78 ± 1.21	3.48 ± 0.25	2.35 ± 0.00	4.68 ± 0.24	0.11 ± 0.01	5.280 ± 0.38	14.17 ± 1.21	0.79 ± 0.02
WHO	6.5–8.5	20–32	5	500	2500b)	NS	NS	NS	6.67b)	250b)	250	50

^a)^ Data are average of triplicates ± standard deviations (SD); nd—not detected; NS—no standard. Water standards (maximum acceptable concentration) of the WHO—World Health Organization (2011). Temperature (Temp), total dissolved solid (TDS), electrical conductivity (EC), dissolved oxygen (DO), biochemical oxygen demand (BOD_5_), chemical oxygen demand (COD), phosphate (PO_4_), sulfate (SO_4_), chloride (Cl^−^), and nitrate (NO_3_)

^b)^ MAC (98/93/EU directive).

The temperature profile of the rainwater during the study regime was statistically significant (*p* < 0.01) and ranged from 27.10 ± 0.98 to 30.35 ± 0.18 °C. The temperatures recorded from the collected rainwater samples conform to the WHO recommended range of 20–32 °C, presenting no risk to individual health when used domestically. Temperature affects the biochemical and chemical reactions that occur in water bodies; elevated temperature allows the toxicity of heavy metals to increase and alleviates living organism's sensitivity to toxic substances.^46^ The turbidity of the rainwater samples was within the range of 1.05 ± 0.01 to 7.24 ± 2.15 nephelometric turbidity unit (NTU). Drinking water legislation recommends that turbidity should not impart any unusual change, and it should be acceptable to consumers. The WHO^40^ stipulates a turbidity level of 5 NTU, and household sites 3, 4, and 6 did not meet the WHO recommended standard (Table 1). Water with high turbidity has been associated with pathogenic microorganisms. When water volumes are increased and stirred with suspended materials, turbidity increases. Hence, high turbidity for a short time may not be as much of a risk as low turbidity that continues for a lengthy time. There were no significant differences in the turbidity levels across the sampled household sites. This may be because the suspended solids easily settle with time at the bottom of the rainwater system reservoir.

The dissolved oxygen (DO), biochemical oxygen demand (BOD), and chemical oxygen demand (COD) values ranged between 3.15 and 5.21, 1.12 and 2.90, and 3.87 and 30.26 mg L^−1^, respectively, as shown in Table 1. DO is essential for maintaining the oxygen balance in water ecosystems. DO concentrations were not significantly different when compared to different sampling points in the study, most likely due to rainfall that resulted in high water volumes. BOD is useful for determining the degree of biological‐related pollution in water systems, which has an adverse effect on water quality. There was a significant difference (*p* < 0.05) in the DO, BOD, and COD of the collected rainwater samples. Low COD signifies that the concentration of biological materials or organic components in the collected rainwater is relatively low. In general, COD indicated the level of oxygen that will be utilized in the oxidation of biological material existing in the water.^47^ COD is closely related to BOD, but it is less widely used than BOD for monitoring of water bodies. The WHO does not specify acceptable guidelines for DO, BOD, and COD.^40^ Major biological activities are highly dependent on pH, organic matter, dissolved oxygen, nutrients, and trace metals. Temperature and pH affect the ability of bacteria to survive in an environment.^46^

The values of the total dissolved solids (TDS) for the rainwater were within safe and allowable limits. TDS of 600 mg L^−1^ or less is generally acceptable for drinking water, but it becomes increasingly and significantly unacceptable when TDS values exceed 1000 mg L^−1^.^25^ Conductivity estimates the overall quantity of dissolved ions or the overall liquefied salts in the water. Electrical conductivity (EC) values were generally low in the rainwater samples (Table 1). EC values lower than 100 μS cm^−1^ signify a low mineralization of harvested rainwater.^48^ Low levels of conductivity in the harvested collected rainwater during the study regime suggest that the harvested rainwater was not charged by dissolved salts or other impurities from the roof, gutters and pipes. Electrical conductivity and TDS vary significantly (*p* < 0.05) between the sites. In general, electrical conductivity had lower values than phosphate (PO_4_), sulfate (SO_4_), nitrate (NO_3_), and chloride (Cl^−^) in the rainwater samples (*p* < 0.05). These attained standards were much lower than the projected recommended guidelines for drinking water (Table 1). The phosphate, sulfate, nitrate, and chloride results (Table 1) in rainwater samples from all the studied household sites (Table 1) revealed no significant differences (*p* > 0.05), which suggested a lack of a role for the parameters from the harvested rainwater, and the slight differences recorded are likely due to the different sampling sites. Nitrates and phosphates have the potential to promote algal growth in the rainwater storage system. The result of nitrates observed in the harvested rainwater samples was found to range from 0.29 ± 0.02 to 0.96 ± 0.04 mg L^−1^, which is lower than the WHO stipulated permissible limit of 10 mg L^−1^ for drinking water. The level of phosphates in all harvested rainwater samples assessed was still lower than the maximum permissible limit of 2 mg L^−1^ set by the European Economic Community standard. Ecological elements coupled with anthropogenic activities are significant factors that affect rooftop harvest rainwater quality, consequently resulting in quality differences for the different locations.

### 2.2 Indicators of Heavy and Light Metals

The analysis of indicators for heavy and light metals in the rainwater samples revealed that Zn (0.13–3.24 mg L^−1^), Ca (1.32–45.6 mg L^−1^), Na (0.10–0.90 mg L^−1^), and K (0.10–3.90 mg L^−1^) (**Table** **2**) were detected below the recommended thresholds. However, zinc was slightly above the MAC level at site 9 with an average concentration of 3.24 mg L^−1^. There were significant differences among the concentrations of Zn, Cu, Ca, and K (*p* < 0.05) across household sites. The heavy metals copper (0.20–1.00 mg L^−1^), lead (0.01–0.85 mg L^−1^), selenium (0.01–1.90 mg L^−1^), and iron (1.40–8.00 mg L^−1^) had concentrations that were orders of magnitude higher than the MAC, so this water could be detrimental to the environment and health of consumers. The quality of the rooftop used to harvest rainwater may contribute to dust contamination in the water. An unpaved road runs through the study area. Vehicles pass consistently on the road, leading to dust particles settling on the rooftops. Similar observations have been observed in other developing countries (South Africa). Nevondo and Cloete^48^ conducted an analysis of the broad‐spectrum quality of harvested rainwater and found it undesirable for potable or domestic uses. Reports from Zhu et al.^43^ and Sazakli et al.^24^ also found that collected rainwater is unacceptable for potable purposes without treatment.

**Table 2:** Heavy metal concentrations of the collected rainwater samples

Sample location	Heavy metals variables (mean ± SD)a)									
	Copper [mg L^−1^]	Iron [mg L^−1^]	Cadmium [mg L^−1^]	Zinc [mg L^−1^]	Calcium [mg L^−1^]	Cobalt [mg L^−1^]	Selenium [mg L^−1^]	Sodium [mg L^−1^]	Potassium [mg L^−1^]	Lead [mg L^−1^]
Site 1	0.27 ± 0.04	5.60 ± 0.15	nd	0.35 ± 0.01	45.6 ± 2.15	nd	0.09 ± 0.0	0.50 ± 0.01	0.50 ± 0.01	0.67 ± 0.11
Site 2	0.23 ± 0.01	1.40 ± 0.12	nd	0.37 ± 0.01	21.6 ± 1.50	nd	0.04 ± 0.0	0.80 ± 0.02	1.00 ± 0.25	0.61 ± 0.20
Site 3	0.28 ± 0.01	6.80 ± 0.25	nd	0.30 ± 0.0	34.8 ± 1.91	nd	0.04 ± 0.0	0.30 ± 0.01	0.70 ± 0.11	0.58 ± 0.15
Site 4	0.31 ± 0.02	7.90 ± 0.14	nd	0.63 ± 0.02	22.8 ± 0.82	nd	0.03 ± 0.0	0.20 ± 0.0	0.20 ± 0.0	0.57 ± 0.18
Site 5	0.28 ± 0.01	7.80 ± 0.58	nd	0.25 ± 0.0	4.2 ± 0.25	nd	1.00 ± 0.0	0.10 ± 0.0	1.50 ± 0.18	0.63 ± 0.21
Site 6	0.28 ± 0.02	3.80 ± 0.90	nd	0.30 ± 0.0	37.2 ± 0.50	nd	1.00 ± 0.0	0.40 ± 0.0	0.30 ± 0.01	0.69 ± 0.15
Site 7	0.30 ± 0.05	7.90 ± 0.18	nd	0.34 ± 0.01	22.2 ± 1.35	nd	1.90 ± 0.25	0.10 ± 0.0	0.10 ± 0.0	0.74 ± 0.14
Site 8	0.32 ± 0.01	7.40 ± 0.84	nd	0.32 ± 0.02	10.8 ± 0.54	nd	1.40 ± 0.15	0.20 ± 0.0	2.20 ± 0.15	0.77 ± 0.10
Site 9	0.25 ± 0.01	5.00 ± 0.31	nd	3.24 ± 0.52	11.4 ± 0.82	nd	1.80 ± 0.16	0.90 ± 0.15	3.90 ± 0.21	0.85 ± 0.24
Site 10	0.30 ± 0.01	8.00 ± 0.92	nd	0.46 ± 0.02	27.6 ± 1.42	nd	1.70 ± 0.05	0.30 ± 0.01	0.20 ± 0.0	0.82 ± 0.20
Site 11	1.00 ± 0.03	3.39 ± 0.48	nd	0.54 ± 0.01	4.30 ± 0.21	0.21 ± 0.01	1.28 ± 0.02	0.69 ± 0.14	0.82 ± 0.0	0.23 ± 0.10
Site 12	0.40 ± 0.02	3.00 ± 0.52	nd	0.13 ± 0.01	35.09 ± 1.49	0.20 ± 0.0	0.07 ± 0.0	0.60 ± 0.12	2.30 ± 0.15	0.01 ± 0.0
Site 13	0.20 ± 0.01	5.00 ± 0.41	nd	0.24 ± 0.02	12.04 ± 0.35	0.20 ± 0.0	0.01 ± 0.0	0.20 ± 0.0	0.32 ± 0.02	0.28 ± 0.15
Site 14	Nd	5.13 ± 0.32	nd	0.70 ± 0.05	12.59 ± 0.59	0.25 ± 0.01	1.30 ± 0.01	0.51 ± 0.10	3.61 ± 0.28	0.31 ± 0.12
Site 15	0.50 ± 0.02	4.34 ± 0.29	nd	0.21 ± 0.01	16.81 ± 0.12	0.10 ± 0.0	0.02 ± 0.0	1.20 ± 0.21	2.11 ± 0.17	0.45 ± 0.16
Site 16	0.30 ± 0.01	5.38 ± 0.35	nd	0.33 ± 0.05	1.32 ± 0.50	0.10 ± 0.0	0.07 ± 0.0	0.30 ± 0.01	1.42 ± 0.12	0.30 ± 0.10
Site 17	1.00 ± 0.13	5.07 ± 0.48	nd	0.29 ± 0.0	9.10 ± 0.48	0.20 ± 0.01	0.30 ± 0.02	0.51 ± 0.10	3.68 ± 0.91	0.51 ± 0.14
Site 18	0.83 ± 0.01	5.10 ± 0.21	nd	0.29 ± 0.0	11.10 ± 0.53	0.10 ± 0.0	1.31 ± 0.01	0.32 ± 0.01	0.41 ± 0.01	0.32 ± 0.01
Site 19	1.00 ± 0.05	6.02 ± 0.42	nd	0.23 ± 0.02	21.01 ± 0.62	nd	0.46 ± 0.05	0.41 ± 0.01	0.61 ± 0.02	0.41 ± 0.02
Site 20	0.60 ± 0.02	3.89 ± 0.012	nd	0.26 ± 0.01	2.24 ± 0.02	0.01 ± 0.0	0.02 ± 0.0	0.59 ± 0.11	0.54 ± 0.0	0.59 ± 0.12
WHO	0.05b)	0.3b)	0.003	3	75b)	NS	0.04	100	20b)	0.01

^a)^ Values are the means of triplicates ± standard deviations (SD); nd—not detected; NS—no standard. Water standards (maximum acceptable concentration) of the WHO—World Health Organization^40^

^b)^ WHO.^58^

Cadmium was found to be below the detectable limits for all rainwater samples at the study area (MAC limit 0.003 mg L^−1^) (Table 2). Cobalt was not detected at sites 1 to 10 and 19, but it was detected at very low concentrations at sites 11 to 18 and 20 (Table 2). The WHO^25^ guidelines recommend that no cobalt levels are permissible. In general, some Fe, Ni, Mn, Zn, and Cu are vital micronutrients for the metabolic activities of animals and plants. Iron (Fe) concentrations in drinking water are normally less than 0.3 mg L^−1^. The minimum daily requirement for iron depends on various factors, with the range being between 10 and 50 mg d^−1^. The lethal dose of iron is ≈200–250 mg kg^−1^ of body weight, but death has occurred after ingestion of doses as low as 40 mg kg^−1^ of body weight. Studies have shown that excess iron can result in hemorrhagic necrosis and sloughing of mucosa areas in the stomach.^40^ Other elements such as Cr, Co, Pb, and Cd have no defined physiological function.^49^, ^50^, ^51^ Environmental sources, such as overhanging vegetation, dead leaves, bird and animal feces and atmospheric pollutants, play a role in the contamination of rainwater; however, these factors vary among locations and depend on the architecture of the rainwater harvesting system itself.^52^ Our heavy metal investigation revealed some degree of similarities among the study sites, and these were also similar to previous studies^53^ that observed elevated concentrations of Zn and Cd from metal rooftops. Higher levels of lead and zinc have been found in rainwater collected via corrugated iron sheets and galvanized iron roofs.^54^, ^55^ This same trend occurred in our study, as cadmium and zinc were not detected, but lead was detected. These results suggest that high lead levels could be associated with plastic storage tanks and underground concrete reservoirs. Lead may have emerged from flashing on the rooftops.^53^, ^56^ The lead concentration in our study is comparable with findings from industrial and urban areas. The different types of rainwater reservoirs and rooftops may be a factor in the variation of water quality.^57^ Continuous use of harvested rainwater, driven in part by water scarcity, can greatly elevate human exposure to these heavy metals. Heavy metals, such as lead, can have extreme consequences on human health. The effect of lead and other metals on public health has been broadly investigated and is regularly reviewed by international organizations, such as the World Health Organization.^58^ Consumption of these nonessentials metals, even at minute concentrations, affects bone and causes neurological diseases, renal dysfunction, cardiovascular disease, and various cancers.^59^ Assessments of rooftop harvested rainwater quality in our study are relevant to the safety of drinking water and its implication for human health.

## 3 Conclusion

The quality of rooftop harvested rainwater sampled in Ugbihioko, a rural community near Benin City, revealed that some parameters were within the permissible limit recommended by the World Health Organization's Potable Water Guidelines.^40^ Roof harvested rainwater quality appears to be linked to the location of the rooftop harvested rainwater systems. In some instances, environmental factors have a negative effect on rooftop harvested rainwater quality, as was observed in the present study. Public education of the hazards associated with the consumption of untreated rooftop harvested rainwater is needed. More so, these water systems can be reservoirs for pathogenic microbes that are harmful to human health. Advanced studies on rooftop harvested rainwater are needed to analyze the role of roofing materials and collection systems on the variability of heavy metal concentrations. Water quality treatment of rooftop harvested rainwater in homes that rely exclusively on this water for drinking and domestic uses is highly recommended.

## 4 Experimental Section

**Figure 1:**
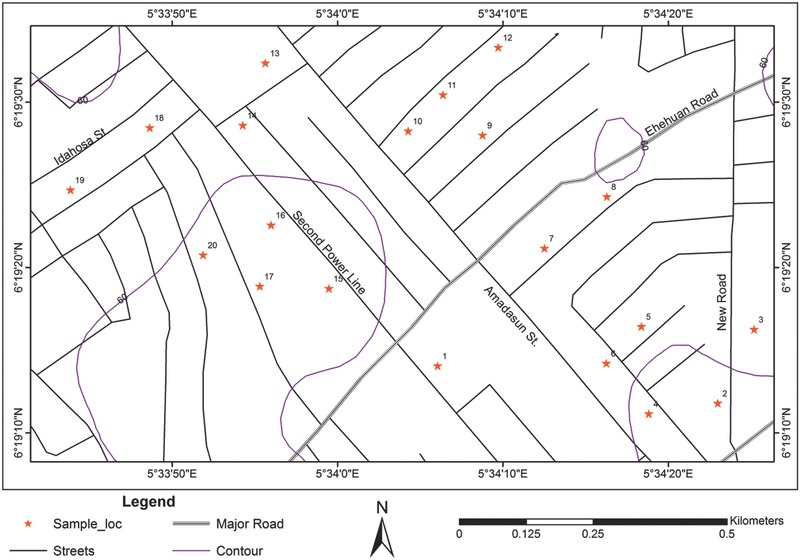
Map of the sampling distribution across the study area. The specific households sampled are indicated by red stars.

*Study Area*: The quality of harvested rainwater was assessed among 20 homes with rainwater harvesting systems located in Ugbihioko, a suburban community in Benin City, Edo State, Nigeria (**Figure** **1**). Ugbihioko is located on the upper Ekehuan road in the Egor Local Government Area of Edo State at latitude 06°19.2341′ and longitude 05°34.10094′. The community is bounded by four other communities: Uzebu, Utagban, Evbuotobu, and Oghede. Residents of Ugbihioko are predominantly farmers and small traders, although some are civil servants. The main water source in the community is harvested rainwater collected from home rooftops into concrete or plastic storage units. Although some inhabitants have recently constructed boreholes, some households still use the traditional means of water collection (rainwater). The sampling regime occurred from April 2015 to September 2015, when rainwater samples were collected at each home concurrently. The wet season in Benin City occurs from April to October, while the dry season occurs from November to March. Rainfall is usually high intensity and occurs frequently with a cessation in August. In general, the mean annual rainfall ranges from 500 to 2780 mm. The mean annual temperature lies within the range of 24–33 °C.^60^

*Study Site*: Although each household that participated in the quality assessment was unique in some respects, the surveys showed that some features were common among households. The households all used concrete or plastic as their rainwater storage material. The home rooftops were made of corrugated iron sheets. Most households used harvested rainwater for all of their household water needs, including drinking, cooking, bathing, laundry, toilet flushing, and other outdoor use.^10^

*Sample Collection*: Rainwater samples were collected aseptically from the rainwater harvesting system. A total of 120 samples were collected from 20 independent households each month between April 2015 and September 2015. Samples of rainwater were collected from the storage reservoir into a standard 1 L sample bottle. Prior to collection, sample bottles were sterilized in an autoclave at 121 °C at 100 kPa [(15 pounds of pressure per square inch (psi)] for 15 min. Before collection of the water sample, the sampling bottles were rinsed with nitric acid and then rinsed with rainwater before being filled with the sample. Each month, all of the samples were collected on the same day to minimize disparity among samples. Samples were placed in cooler boxes and transported to the laboratory for analyses within 4–6 h after collection.

*Physicochemical Analysis*: All instruments and apparatuses were properly calibrated and checked to ensure that they were in accordance with the manufacturer's conditions. The following physicochemical parameters were analyzed in the quality assessment regime: pH, DO, temperature, TDS, BOD_5_, turbidity, EC, COD, SO_4_, PO_4_, Cl^−^, and NO_3_. Temperature, pH, EC, and DO were determined on site using the respective electrodes (Hanna Instruments, version HI 9828). TDS concentrations were measured using a standard gravimetric method.^61^ Turbidity was measured on the field with the aid of a turbidimeter (HACH Company, model 2100P). The levels of PO_4_, SO_4_, Cl^−^, NO_3_, BOD_5_, and COD were determined using a standard photometric method.^61^

*Heavy and Light Metal Indicators*: Indicators for heavy and light metals, such as iron (Fe), copper (Cu), zinc (Zn), cadmium (Cd), calcium (Ca), cobalt (Co), selenium (Se), sodium (Na), potassium (K), and lead (Pb), were tested. Metal concentrations were determined using an atomic absorption spectrophotometer (Perkin Elmer Analyst 800 series Graphite Furnace AA), in accordance with the analytical procedures outlined in the Standard Methods for the Examination of Water and Wastewater^61^ using nitric acid digestion.

*Statistical Analysis*: Data generated were evaluated using a statistical analysis system (SAS) (SAS version 8, SAS Institute, Cary, NC). Descriptive statistics were used to express data as the means ± standard deviations. Analysis of variance was used to identify significant differences (*p* < 0.05 or *p* < 0.01).

## Conflict of Interest

The authors declare no conflict of interest.
